# Contemporary Management of the Failing Fontan

**DOI:** 10.3390/jcm13113049

**Published:** 2024-05-22

**Authors:** Prashanth Venkatesh, Hans Gao, Islam Abudayyeh, Ramdas G. Pai, Padmini Varadarajan

**Affiliations:** 1 Smidt Heart Institute, Department of Cardiology, Cedars-Sinai Medical Center, Los Angeles, CA 90048, USA; prashanth.venkatesh@cshs.org (P.V.); gao@cshs.org (H.G.); 2 Jerry L. Pettis VA Hospital, Loma Linda, CA 92357, USA; iabudayyeh@mac.com; 3 California University of Science and Medicine, Colton, CA 92324, USA; ramdaspai@yahoo.com

**Keywords:** congenital heart disease, Fontan circulation, Fontan failure, single ventricle physiology

## Abstract

Adult patients with congenital heart disease have now surpassed the pediatric population due to advances in surgery and improved survival. One such complex congenital heart disease seen in adult patients is the Fontan circulation. These patients have complex physiology and are at risk for several complications, including thrombosis of the Fontan pathway, pulmonary vascular disease, heart failure, atrial arrhythmias, atrioventricular valve regurgitation, and protein-losing enteropathy. This review discusses the commonly encountered phenotypes of Fontan circulatory failure and their contemporary management.

## 1. Introduction

Congenital heart disease (CHD) occurs in about 1% of live births [1]. The advances made in the treatment and management of patients with congenital heart disease have led to significant improvement in prognosis and improvement in survival with >97% reaching adulthood [2,3,4]. Despite these advances, adults with congenital heart disease (ACHD) encounter many complications, such as heart failure, diabetes mellitus, anatomic sequelae of complex surgeries, arrhythmias, stroke, cancer, and premature death [5,6,7,8,9]. Palliative and reparative surgeries are most often carried out in childhood, with many requiring further interventions with age, influencing prognosis in ACHD patients [10,11]. As such, even patients with the most complex pathology routinely live into adulthood. This has resulted in ACHD patients outnumbering pediatric CHD patients in North America [12].

## 2. Single-Ventricle Patients Have the Highest Mortality

The survival rate of patients with complex CHD is lower than that of the general population. Muller et al. have shown, in their study spanning over 15 years of follow-up, that mortality is dependent on the severity of CHD [13]. In the same study, they showed that ACHD patients had a 16-fold higher mortality rate than expected for age-matched healthy controls. Compared to patients with mild-to-moderate forms of CHD, those with severe CHD have increased mortality [13,14,15], with the single-ventricle subgroup having the worst long-term survival, with twice the mortality as those with complex biventricular CHD complexity. Constantine et al. showed that an increasing number of Fontan patients are surviving to the fourth decade of life and beyond, and that older patients with a Fontan circulation have substantial mortality, with heart failure being the most common cause of death. Despite improvements in medical and interventional therapy, 10-year mortality remains high after the age of 35 years, with ~1 in 10 patients dying within 5 years and 1 in 5 patients dying within 10 years [16].

## 3. Fontan Palliation and Why

**Figure 1:**
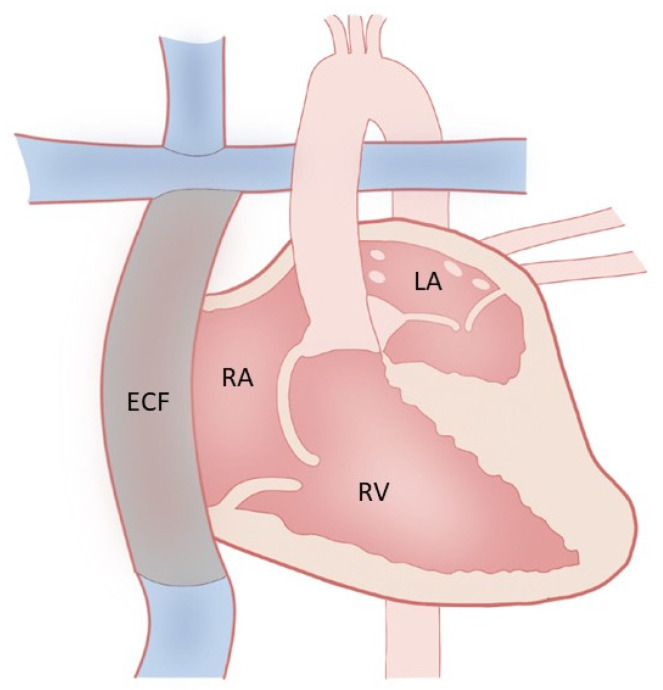
Hypoplastic left ventricle with extracardiac Fontan.

Fontan palliation (Figure 1) is performed in patients with complex congenital heart disease, especially in those with single-ventricle physiology (such as hypoplastic left heart syndrome, tricuspid atresia, and double-outlet right ventricle, double-inlet right ventricle). In these conditions, there is a single dominant and functioning ventricle, with the other being hypoplastic. After a series of surgeries starting in early infancy, the final Fontan operation results in a total cavopulmonary anastomosis, with systemic venous return from both the inferior vena cava (IVC) and superior vena cava (SVC) passively flowing directly into the pulmonary arteries, thus bypassing the heart altogether.

## 4. Fontan Physiology and Failure

In contrast to a normal heart with two synchronized ventricles and the pulmonary and systemic circuits working in series, the Fontan circulation does not have a subpulmonic ventricle, which is associated with elevated pressures in the vena cavae, with non-pulsatile flow in the pulmonary arteries and a mild reduction in systemic cardiac output [17]. In a normal heart, the subpulmonic ventricle’s contribution of blood flow to the pulmonary arteries allows a normal central venous pressure (CVP). Conversely, in a Fontan circulation, CVP has to be greater than the pressure in the pulmonary arteries to allow passive flow down a pressure gradient and is hence in the 12–14 mmHg range. This concept is well known as the Fontan Paradox. Cardiac output is affected in the Fontan circulation due to passive cavopulmonary flow without the contribution of a subpulmonic ventricle. The system is unable to deliver normal blood volume to the pulmonary circuit, leading to a low preload of the systemic ventricle and a low stroke volume with chronic volume depletion of the systemic ventricle despite a paradoxically elevated CVP. The passive filling of the pulmonary arteries is unable to be augmented sufficiently with exercise, leading to a chronically decreased functional capacity in most patients [17].

Fontan circulatory failure (FCF) constitutes a broad, term which describes dysfunction of the Fontan circulation, affecting a patient’s ability to carry out daily life activities, as per the consensus statement by Alsaied et al. [18,19]. Table 1 shows some of the common phenotypes of Fontan circulatory failure.

## 5. Systolic Dysfunction

Ventricular function, which tends to be normal at the time of Fontan surgery, usually worsens over time. In one major study, it was shown that Fontan patients had heart failure after a median of 18.1 years after Fontan completion, and over 40% had systolic dysfunction. It’s also known that about 50% of patients with Fontan listed for heart transplantation end up having systolic dysfunction [20]. The actual cause of this systolic dysfunction is not well known. Hypoplastic left heart syndrome comes with the highest risk for systolic dysfunction, as the right ventricle is designed to function as a systemic ventricle [21]. Exposure to chronic hypoxemia probably predisposes to ventricular dilatation and dysfunction, though with initial improvement soon after surgery [22]. Myocardial fibrosis, abnormal fiber orientation causing mechanical dyssynchrony, and chronic low preload with lower fiber stretch and contractility are some of the speculated causes of systolic dysfunction [22,23,24,25]. The loss of normal descent of the atrioventricular (AV) valve(s) toward the apex also can cause a loss of suction force and further reduction in preload. This suction force in the normal heart is important to drive the blood to the pulmonary circuit and becomes even more important in a Fontan patient due to the absence of the subpulmonic ventricle and reliance on a gradient from the systemic veins to the pulmonary venous atrium to drive antegrade flow [26].

## 6. Diagnosis

Echocardiography is the standard first-line tool used to delineate anatomy and assess ventricular function. Recent literature has shown that cardiac magnetic resonance imaging (MRI) may be more useful to diagnose systolic dysfunction in Fontan patients, especially with a systemic right ventricle. Most ACHD centers use both echocardiography and MRI as complementary tools to identify systolic dysfunction [27]. Needless to say, patients with lower end-diastolic volumes had a better prognosis in a large study. Furthermore, increased ventricular end diastolic volume of >156 mL/BSA, combined with lower circumferential strain was predictive of early death or transplant [28]. Cardiac MRI can also help in the evaluation of myocardial fibrosis, but further research is needed to evaluate its correlation with ventricular function and outcomes [29]. Dobutamine stress MRI can also be useful in evaluating contractile reserve, the lack of which portends poor prognosis in this population [30].

## 7. Diastolic Dysfunction

Diastolic dysfunction is increasingly recognized as a major pathology in Fontan patients, and particularly in >80% of patients with a single right ventricle [22,31]. Diastolic dysfunction can be caused by factors such as dyssynchrony, abnormal ventricular geometry, fibrosis, increased wall stress, hypoxemia, and increased volume load prior to Fontan surgery [21]. Studies have described the prolongation of isovolumetric relaxation time, which in turn leads to a decrease in early rapid filling [31]. This can then lead to a reduction in ventricular compliance, impacting late-diastolic flow and increasing filling pressures [32]. Due to a requirement for passive flow in Fontan patients, higher filling pressures are not well tolerated. Higher filling pressures cause higher atrial pressures, with a reduced gradient for flow into the pulmonary circulation, in turn leading to reduced preload and stroke volume [33]. Notably, diastolic dysfunction can also be caused by lifestyle- related factors.

## 8. Diagnosis of Diastolic Dysfunction

Diagnosis of diastolic dysfunction by echocardiography remains to be conclusively validated in patients with a Fontan circulation. Doppler indices, specifically the E/e’ ratio, appeared to correlate with invasive measurement of ventricular filling pressure in a single study by Miranda et al.; however, simultaneous echocardiographic-catheterization correlation studies are required to validate the diagnostic utility of echocardiographic measurements for diastolic assessment in this patient population [33].

## 9. Atrioventricular Valve Regurgitation (AVVR)

Annular dilatation, leaflet prolapse, tethering, dysplasia or abnormal sub-valvular apparatus can lead to AVVR in Fontan patients [34]. Atrioventricular valve failure (AVVF) needing intervention, such as valve repair or replacement, occurs in about 7% of Fontan patients at 5 years, increasing to 12% and 21% at 10 and 20 years, respectively [35]. Systemic right ventricle, as seen in hypoplastic left heart syndrome has been shown to be an independent risk factor for AVVR [35]. Fontan patients tolerate AVVR very poorly, due to the vicious cycle of ventricular dilatation, worsening dysfunction and more AVVR, as well as increasing atrial pressure which in turn reduces pulmonary venous filling by blunting the venous pressure gradient and thus reduces cardiac output.

## 10. Diagnosis of AVVR

Diagnosis of AVVR can be evaluated with multimodality imaging. Traditional two-dimensional echocardiography (2DE), though used routinely, may not be able to accurately evaluate the etiology of AVVR. Three-dimensional echocardiography (3DE) is considerably better at evaluating the valve and sub-valvular apparatus [36]. Cardiac MRI can be useful to detect flow and quantify the degree of AVVR as well as ventricular function and volume, though it is unable to be used universally due to the presence of non-compatible pacing systems or abandoned pacing leads.

## 11. Lymphatic Insufficiency

Fontan patients will develop leakage of chyle into bronchial, pleural, peritoneal, or other intestinal cavities due to increased lymphatic congestion brought on by elevated central venous pressures. This leads to the development of chylous ascites, plastic bronchitis (PB), and protein-losing enteropathy (PLE) [22]. In Fontan patients, protein-losing enteropathy is seen in 4–13% [37,38], while PB is seen in 2–4% [39]. A classification system exists that describes the neck and thoracic lymphatic abnormalities, which then equates to adverse surgical outcomes, mortality, and morbidity [40]. Lymphatic insufficiency is known to cause edema, immune deficiency, malnutrition, chronic cough, respiratory distress, hypoxemia, and finally death [22], though with improved outcomes in the past decade [14].

## 12. Diagnosis of Protein-Losing Enteropathy and Plastic Bronchitis

Diagnosis of PLE is determined with stool measurement of alpha-1-antitrypsin (A1AT) or by nuclear scintigraphy using technetium-99m-labeled albumin. The A1AT values are a spot A1AT level of >54 mg/dL, >27 mL/24 h without diarrhea, or >56 mL/24 h with diarrhea. [19,41]. Dynamic contrast MRI lymphangiography and lymphoscintigraphy are some imaging modalities that can be used to diagnose PLE [40]. Serum albumin concentration can be checked on a routine basis to evaluate for loss of albumin, which can then point to a diagnosis of PLE [22]. It is important to emphasize that the PLE can be subclinical, and hence, periodic laboratory evaluation is key.

PB is diagnosed clinically when patients expectorate mucoid casts of the pulmonary tree. Dynamic contrast MRI lymphangiography can be additionally helpful.

## 13. Arrhythmia

Arrhythmias are a common occurrence after Fontan surgery, with sinus node dysfunction, iatrogenic AV block, and supraventricular tachycardias being the most common [22]. Ventricular tachycardias occur less commonly in Fontan patients [42]. It has been estimated that about 10% of patients will need pacemaker implantation, especially to maintain atrioventricular synchrony and improved hemodynamics [43]. Iatrogenic conduction system injury, loss of arterial supply to the sinus node, suture lines affecting conduction, atrial dilatation, and tissue fibrosis from cyanosis are some of the causes of arrhythmias [22] and can cause a reduction in cardiac reserve. They can also cause heart failure, which can lead to an increased need for transplants [31].

## 14. Diagnosis

With frequent occurrence of arrhythmia in Fontan patients, ambulatory electrocardiographic monitoring is recommended every 1–2 years in adolescents and adults based on physiologic stage. Ambulatory electrocardiographic monitoring should additionally be obtained in patients experiencing signs and symptoms of Fontan failure [22].

## 15. Medical Treatment (Table 2)

There is scarce data regarding medical treatment for FCF. Most of the medical treatment has been extrapolated from heart failure therapy in biventricular circulations. While diuretic therapy helps in alleviation of congestive symptoms of both systolic and diastolic heart failure, it can also lead to a fall in cardiac output by preload deprivation and also precipitate renal dysfunction [22]. Beta blockers (BB) and renin-angiotensin-aldosterone (RAAS) inhibitors, which are used as a cornerstone of therapy in acquired heart failure in adults with biventricular circulations, can be used in Fontan failure, albeit with very scant data [31,44]. The harmful effect of beta blocker use in failing Fontan was shown in a randomized trial [45], while use of angiotensin-converting enzyme inhibitors (ACEIs) did not improve ventricular function in another small trial [46]. The beneficial role of high-dose spironolactone in a select subset of FCF patients with PLE has been shown in some small studies [47,48]. In our experience, mineralocorticoid receptor antagonists (MRA) can be effective due to the underlying hepatic dysfunction that is seen in most patients with Fontan failure.

## 16. Treatment of Increased Pulmonary Vascular Resistance (PVR)

Increased PVR, defined in Fontan patients as a PVRi > 2 WUm2, can result in Fontan failure and can occur either due to systemic ventricular failure or chronic atrial hypertension due to arrhythmia/AVVR, or result from a pre-capillary etiology such as smoking, obesity, and pulmonary pathology [19]. Drugs such as phosphodiesterase type 5 inhibitors (PDE5s) like sildenafil can be of benefit in reducing PVR, decreasing arterial elastance and ventricular end-systolic and end-diastolic elastance, and improving preload. Sildenafil has shown improvement in exercise hemodynamics and capacity and improved ventricular performance in small studies [49], but in the larger FUEL trial, udenafil did not show improvements in peak oxygen consumption, though there was improvement in other measures, including work rate and ventilatory efficiency [50].

Pulmonary vasodilation may be of benefit in a subset of patients with lymphatic failure [51]. Another available option in PLE is the use of midodrine which is an alpha 1 receptor agonist, which may improve the tone of lymphatic vessel smooth vessels, has shown improvement of symptoms leading to delay in heart transplant [52]. Treatment with oral octreotide and budesonide have been tried but may not help delay time to transplant [53].

## 17. Treatment of Arrhythmias

Arrhythmias can have detrimental effects on ventricular function in Fontan patients. Antiarrhythmic drugs can be used but are limited by side effects in this young population. Their use can lead to a higher rate of recurrence compared to that of catheter ablation, which is still the preferred treatment for these patients, especially if a congenital electrophysiologist is accessible [22]. For patients needing a pacemaker, employing a mode to help restore atrioventricular synchrony is beneficial [42,54]. Dual-site epicardial pacing has been employed in patients with heart block or with ventricular dyssynchrony, with recent data showing improvement of ventricular function and New York Heart Association (NYHA) classification [55]. Fontan patients should be encouraged to undergo exercise therapy, which may help improve peak oxygen uptake and cardiac output along with quality of life, as shown in some small series [56]. In cases of sinus node dysfunction with intact AV nodal function, transvenous atrial lead implantation into the posterior atrial wall through the left pulmonary artery has been described and can be utilized, especially if epicardial lead placement involves a median sternotomy [57].

## 18. Advanced Therapies for Heart Failure

### Use of Inotropes

Patients who have undergone Fontan palliation and experience low-output failure compromising end-organ perfusion may benefit from intravenous inotropes. Data are lacking regarding the use of inotropes in these patients, which is hence based on and extrapolated from patients with heart failure with biventricular circulation [58]. Milrinone, which is a phosphodiesterase-3 inhibitor, has lusitropic, inotropic, and vasodilatory properties, making it attractive for the treatment of a failing Fontan patient [59]. Similarly, milrinone, when used at the time of initial repair, has been shown to improve hemodynamics, but similar data is lacking in the adults with Fontan failure [60]. In our experience, milrinone has the most benefit in systolic dysfunction; while it can be used to augment diuresis in Fontan failure with preserved ventricular ejection fraction, care must be taken to not use inodilator therapy in patients with cirrhosis and low systemic vascular resistance. As data are lacking, the use of chronic inotrope in this subset of patients is not recommended but is commonly and successfully used as an inpatient bridge to more definitive therapy like heart transplant [61]. In patients with protein-losing enteropathy (PLE), dopamine has been shown to be helpful in producing remission by its action on lymphatic receptors [62].

## 19. Mechanical Circulatory Support

**Figure 2:**
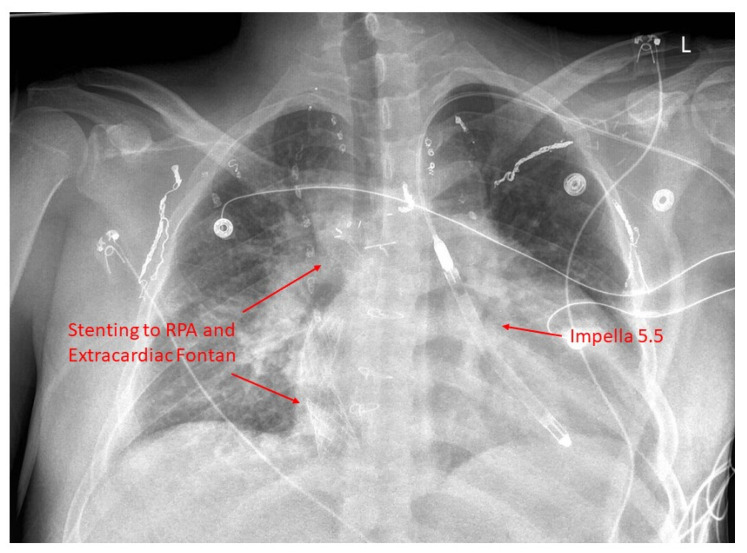
Chest X-ray of a patient with hypoplastic left heart and extracardiac Fontan with temporary mechanical circulatory support as bridge to heart transplant.

With adults with CHD outnumbering children with CHD, the number of patients with Fontan failure is growing. The need for heart transplantation is also increasing, while organ supply is increasingly short, with long wait times on the transplant list driving the need for durable mechanical circulatory support (MCS) (Figure 2). The use of MCS will not address the underlying cause of Fontan failure. Usually, MCS is beneficial for patients with systolic ventricular dysfunction rather than other phenotypes of Fontan failure [63]. MCS is gaining momentum in the failing Fontan patients [64,65]; however, outcomes are worse compared to those with heart failure with biventricular circulation [66,67,68]. The use of MCS is fraught with many challenges, even though the field is evolving and improving. A history of multiple cardiac surgeries in childhood leads to challenging redo sternotomy and dissection. The orientation of the single ventricle may make the positioning of cannulas challenging [68]. Similarly, the single ventricle may have dense trabeculations, making the placement of inflow cannulas difficult and requiring extensive trabeculectomy [63]. Nandi et al., in their paper, have discussed the use of novel approaches such as atrial cannulation and excision of the atrioventricular (AV) valve to enable device placement in smaller patients [69]. Villa et al. were the first to report the use of MCS as a bridge to combined heart and liver transplantation (CHLT) in a 22-year-old man with Fontan circulatory failure [70]. Since the first report, about 400 patients have undergone placement of a ventricular assist device (VAD), with about 40 ending up with CHLT [71]. Similarly, there are a few case reports on the use of the Syncardia total artificial heart and single subpulmonary VAD in Fontan circulatory failure but without wide acceptance [72,73]. There are also reports of using temporary VAD along with Impella support in a limited number of Fontan patients, both adult and pediatric, with cardiogenic shock. [74,75]. This use of MCS may be beneficial as a bridge to recovery or used for longer term support.

## 20. Transplantation

Heart transplantation is considered definitive treatment in the management of Fontan circulatory failure, though this is only performed in select ACHD centers. It is also known that adults with Fontan listed as status 1A for a transplant have higher mortality while waiting on the list compared to those with no CHD [76]. Fontan patients pose a substantial risk for transplantation but with improvements in the recent past [77,78]. Though recent studies have reported a 1-year survival of 89%, which is similar to that of non-Fontan patients, it is not on par with patients who do not have congenital heart disease [77,79]. Patients with Fontan have multiple unique risk factors, such as previous surgeries, sensitization due to prior blood transfusions, and aortopulmonary collaterals. At the time of transplantation, they encounter increased surgical complications, such as increased bleeding, complex dissection due to multiple previous surgeries, longer ischemic times, and rejection [80,81], resulting in significant mortality and morbidity [80].

Serfas et al. have shown increasing age and preserved left ventricular function (LV) as contributors for worse outcomes in adult patients, with preserved LV function causing long-term malnutrition, immunosuppression, and pulmonary problems, resulting in worse outcomes [81]. It has been shown that Fontan patients with successful transplants have shown resolution of PLE [82,83]. Causes of death in Fontan patients after transplantation include infections, early graft failure, rejection, and sudden death, which are similar to those of other heart transplant patients [80]. Patients with Fontan commonly have liver disease, and some undergo CHLT. Sganga et al. compared outcomes in patients with heart transplant (HT) alone to CHLT in a small study of 47 patients (9 CHLT, 38 HT). CHLT patients were older, had worse liver disease, and were likely to be on dual inotrope therapy. Ischemic times were longer in the CHLT group, with similar post-operative complications [84]. They also showed that over a median follow-up of 17 months, overall mortality was 17%; only one patient with CHLT died versus 7 with HT (*p* = 0.64). Patients with HT alone who had cirrhosis pre-transplant had worse outcomes. They concluded that despite being on inotropes and having severe liver disease, CHLT patients have similar outcomes to those with HT alone, with survival benefits in Fontan patients with liver disease. Despite this, more data is warranted, and research is needed in this field of Fontan patients. 

## 21. Catheter-Based Therapies for Fontan Failure

### Arrhythmias

Persistent atrial arrhythmias contributing to heart failure symptoms are very common and often seen in those with atriopulmonary (AP) Fontan surgery. Moore et al., in their study, have shown catheter-based ablation to be an effective modality to reduce arrhythmic burden [85]. They reviewed arrhythmia data in 47 patients with AP Fontan patients. This study showed that all inducible tachycardia was abatable in 62% and at least one includible tachycardia could be addressed in 25%, while, in 13%, none of the tachycardia could be ablated. Cather-based ablation resulted in a reduction of arrhythmia score during a follow-up of 24 months, while 12 patients (29%) needed a repeat ablation procedure, with a mean time of 2.7 ± 3 years in between procedures. Atrial fibrillation is also an emerging arrhythmia in this population, and pulmonary vein isolation has also been successfully described. We anticipate seeing the use of this modality more frequently as the Fontan population continues to age.

## 22. Fontan Pathway Obstruction

**Figure 3:**
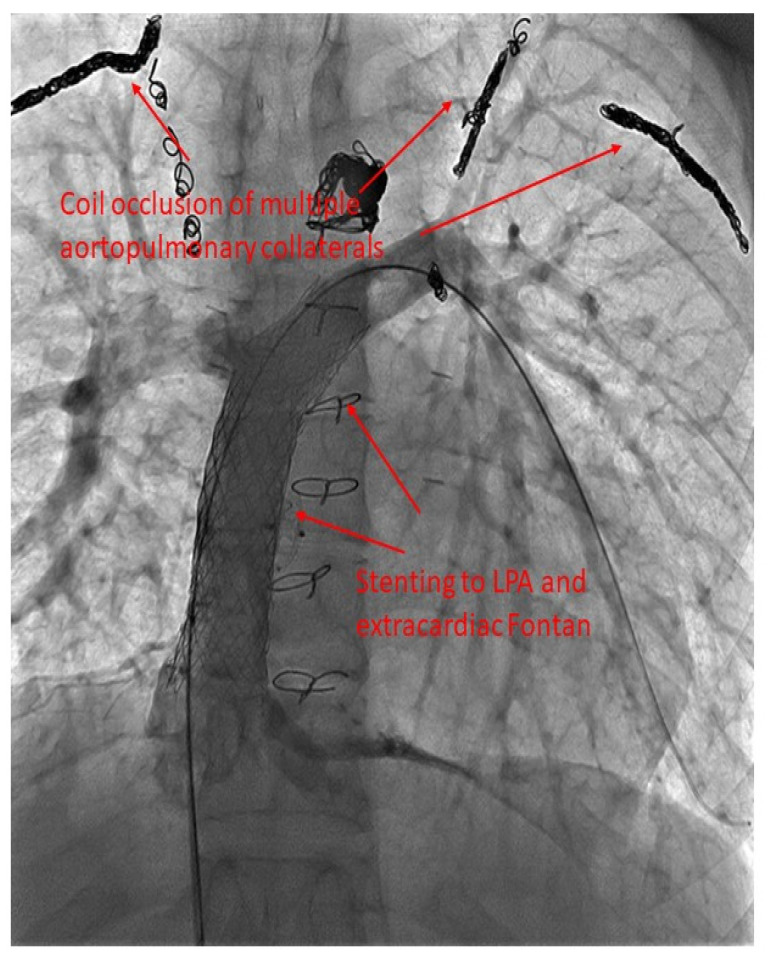
Selective angiography of extracardiac Fontan and left pulmonary artery status post multiple stents.

**Figure 4:**
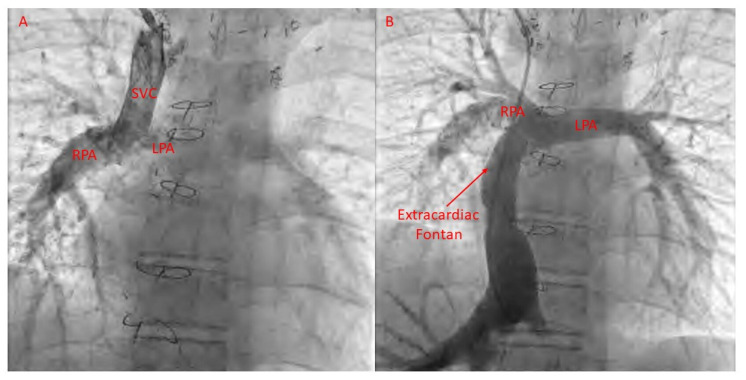
Selective angiography of bidirectional right-sided Glenn shunt (**A**) and extracardiac Fontan (**B**).

Obstruction of the Fontan pathway due to stenoses in the vena cavae, anastomoses, or pulmonary artery is seen in patients with Fontan palliation. Catheter-based relief of obstruction, with placement of bare-metal stents, has been shown to be effective [86] (Figure 3 and Figure 4). Agasthi et al. conducted a retrospective analysis of 26 adult patients who had stent placement for Fontan pathway obstruction [87]. They showed that percutaneous stenting was safe and effective at relieving obstruction and helping with improvement in quality of life. They further demonstrated that, in a few patients, stenting was associated with improvement in markers of portal hypertension, thereby speculating about evidence for improvement in Fontan-associated liver disease with stenting [87]. Further research is needed in this regard.

## 23. Lymphatic Disease

Research to address lymphatic Fontan failure percutaneously is ongoing. Of the various therapies available, thoracic duct interventions and selective lymphatic embolization are being used to treat patients with complications of the lymphatic system [88,89,90,91]. Imaging modalities, such as magnetic resonance lymphangiography (MRL), are useful, with good spatial resolution for imaging both peripheral and central lymphatic systems. Intranodal dynamic contrast MRL (DCMRL) is a recent addition to the imaging armamentarium and has become the modality of choice for studying central lymphatic flow disorders [91]. The technique has good spatial and temporal resolution and involves intranodal gadolinium injection, which is followed by dynamic and static contrast-enhanced MR imaging. This results in quick enhancement of central lymphatic channels, evaluating dynamic flow and static anatomy. Intranodal DCMRL is used for assessment of the central lymphatic system before surgery by the identification of leaks and lymphatic flow disorders, which can then be treated with catheter-based procedures [92,93]. In Fontan patients with lymphatic failure, cardiac catheterization is recommended to evaluate hemodynamics and superior vena cava or pulmonary branch stenosis, which can be reversible. Creation or recreation of Fontan fenestration can be performed in patients with Fontan procedures to alleviate pressure and PLE [92]. Decompression of the lymphatic system can be achieved either with lymphovenous anastomosis (LVA) or surgical or percutaneous thoracic duct (TD) decompression. Selective lymphatic duct embolization, covered stent placement in TD, ethiodized oil lymphatic embolization, liver lymphatic embolization, and TD embolization or ligation serve to exclude abnormal lymphatic channels [92,93].

## 24. Lymphatic Embolization with Ethiodized Oil

Injection of inguinal lymph nodes with ethiodized oil can help in visualization of the central lymphatic system and assist in mechanical occlusion of distal lymphatic channels due to high viscosity and causing sclerosis of the smaller vessels. There is an elevated risk of systemic embolization with this procedure; hence, it is used only in patients without a significant right-to-left shunt [93].

## 25. Thoracic Duct Embolization

Thoracic duct embolization can be used in select patients with chylothorax, chylopericardium, and plastic bronchitis. Transvenous retrograde cannulation of TD helps in accessing the central lymphatic system, with microcatheter placement over a wire into the TD. Embolization is then performed with n-butyl cyanoacrylate endovascular glue, with or without occlusion coils. TD flow obstruction with external compression or transvenous balloon occlusion of the lymphovenous junction at the subclavian vein can help prevent the exit of glue from the TD outlet [89]. It must be noted that this is not the treatment for PLE—the TD, if embolized in the wrong patient, can cause diffuse severe lymphovenous congestion; any decision to embolize the TD should be taken only after multidisciplinary consultation with careful angiography for planning purposes.

## 26. Selective Lymphatic Duct Embolization plus Covered Stent Placement

Advancing a microcatheter into the collateral branches arising from the TD and injecting glue or embolizing with coils and glue can help in selective lymphatic duct embolization. The abnormal vessels can also be directly accessed via a needle and then embolized. In patients with elevated central venous pressure (CVP), selective lymphatic duct embolization is preferred as the TD can be preserved when access for other interventions is needed. In patients with multicompartment lymphatic failure leading to PLE, ascites, plastic bronchitis, or chylothorax, this method is preferred [89].

## 27. Liver Lymphatic Embolization (LLE)

This procedure can be undertaken when there is a leak of contrast into the peritoneum in patients with ascites or contrast leak into the duodenal lumen in patients with PLE, or in the presence of hepatopulmonary connections causing plastic bronchitis or chylothorax. In this procedure, patients undergo endoscopy with visualization of the periampullary region; following this, isosulfan blue dye is injected through a needle in the hepatic lymphatics to confirm contrast leak without any leak from the ampulla. Glue is then injected through the needle that results in liver lymphatic embolization [94]. Long-term outcomes of these procedures are still being actively researched.

## 28. Percutaneous Thoracic Duct (TD) Decompression

In patients with total cavo-pulmonary connection, CVP is elevated, which results in increased fluid production along with impaired drainage, leading to complications such as PLE, plastic bronchitis, chylothorax, ascites, or multicompartment lymphatic failure. TD decompression will reduce afterload and may reduce the formation of abnormal lymphatic networks. While surgical decompression has been practiced as the gold standard, a new percutaneous method to decompress the TD was recently reported by Smith et al. [95].

## 29. Percutaneous Intervention for AV Valve Regurgitation

**Figure 5:**
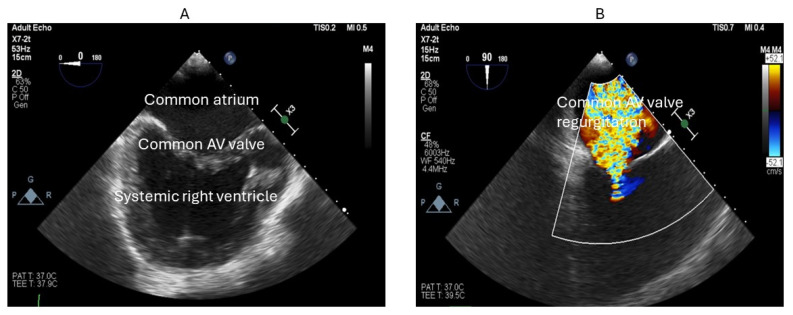
Transesophageal echocardiogram demonstrating an enlarged common atrium with a deformed atrioventricular valve (AV) (**A**) and severe AV valve regurgitation (**B**).

**Figure 6:**
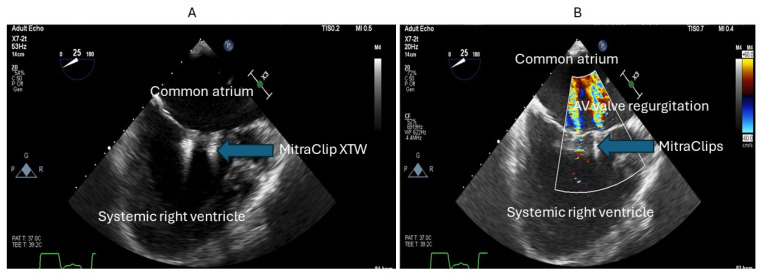
Transesophageal echocardiogram demonstrating two MitraClips XTW (**A**), and an improvement in atrioventricular (AV) regurgitation severity (**B**).

**Figure 7:**
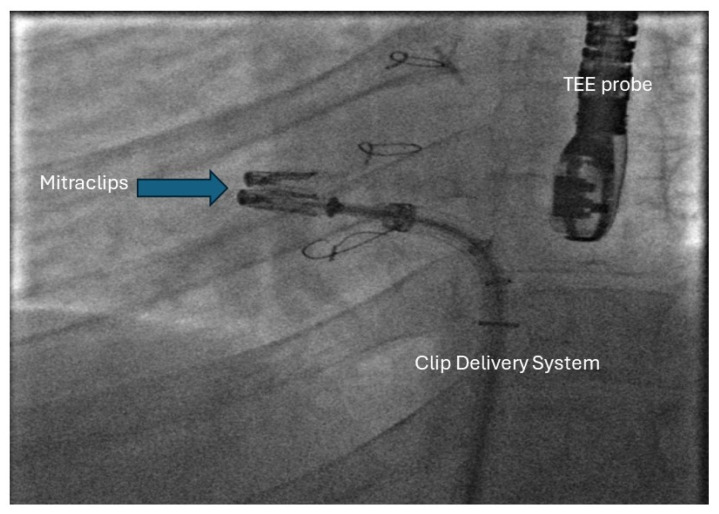
Fluoroscopic image showing the MitraClips in Position.

AVVR is very commonly seen in patients with Fontan circulation, yet the surgical risk for AV valve intervention is high. In such patients, the use of transcutaneous edge–edge repair (TEER) has been explored in isolated case reports in Fontan patients. Access to the systemic atrium could be challenging and may require puncture of the Fontan conduit. However, when the anatomy is suitable, TEER of AV valve regurgitation could be beneficial in these patients. Kumar et al. have reported the use of TEER to treat severe AV valve regurgitation in a patient with double-outlet right ventricle with a Fontan palliation [96] (Figure 5, Figure 6 and Figure 7).

## 30. Collateral Vessels

Collaterals, in the context of the Fontan circulation, are problematic. The two types of collaterals in the Fontan circulation are: (1) aortopulmonary collaterals arising from the branches of the aorta (or the aorta itself) and leading to the pulmonary veins; and (2) venovenous collaterals arising from the central veins and connecting to the pulmonary veins. Aortopulmonary collaterals form in response to hypoxia, while venovenous collaterals form in response to elevated CVP, and decompress the Fontan circuit. Aortopulmonary collaterals increase Fontan pressure due to arterial vessels feeding into the pulmonary circuit, while venovenous collaterals cause hypoxia. Coil embolization of aortopulmonary collaterals can be effectively performed to lower Fontan pressure [97]. Coil embolization of venovenous collaterals may be performed for relief of hypoxia, though this may be counterproductive in patients with elevated Fontan pressure, in whom the collaterals serve as a much-needed pressure pop-off. Poterucha et al. found increased mortality in Fontan patients whose venovenous collaterals were coiled; most of these deaths occurred with elevated Fontan pressures, suggesting that, in such patients, permissive hypoxia should be accepted and these collaterals left unintervened [98].

Of these two collateral types, the aortopulmonary collaterals pose a significantly larger risk during surgery due to the systemic arterial pressure in these vessels and the risk of bleeding, either during chest entry or while coming off the pump after a heart transplant has been successfully anastomosed. These collaterals increase the risk of hemorrhage during cardiac surgery, especially during Fontan transplantation [99]. The practice of prophylactic percutaneous collateral embolization prior to transplantation was studied by Tan et al., who found that this led to a reduction in intraoperative collateral flow in all comers, and a trend towards a reduction of postoperative bleeding complications and reoperations in patients receiving CHLT [100].

## 31. Summary

FCF is a complex, multi-faceted state with evolving therapies. Newer imaging modalities and transcatheter approaches are of great utility in managing Fontan pathway obstruction, lymphatic failure, arrhythmias, and AV valve regurgitation, while either HT or CHLT transplant offers definitive therapy. Further therapeutic options need to be explored in order to improve outcomes in this complex patient population.

## Figures and Tables

**Table 1:** Causes of Fontan Circulatory failure.

Causes of Fontan circulatory failure
Systolic dysfunction
Diastolic dysfunction
Anatomic obstruction
Lymphatic insufficiency/protein-losing enteropathy/plastic bronchitis
Arrhythmia
Atrio-ventricular valve regurgitation

**Table 2:** Contemporary medical therapy for FCF.

Heart failure	Diuretics, BB, RAAS inhibitors, ACEI, MRA
Pulmonary vascular modulators	PDE5 inhibitors, Endothelin receptor antagonists and prostacyclin analogs
PLE	Midodrine, oral budenoside, octreotide
Arrhythmia	Anti-arrhythmics
Conduction abnormalities	Pacing with restortion of AV synchrony, resynchronization therapy
Quality of life	Exercise therapy
